# Between legal rights and lived realities: Roma Women’s experiences of abortion care analyzed using the WHO conceptual framework for abortion care

**DOI:** 10.1177/17455057261458329

**Published:** 2026-06-08

**Authors:** Sara Rivenes Lafontan, Frode Eick, Karoline Hasle Einang

**Affiliations:** 1 Institute of Nursing and Health Promotion, OsloMet - Oslo Metropolitan University, Oslo, Norway; 2 155319 Lovisenberg Diaconal University College, Oslo, Norway; 3 6305 University of Oslo, Oslo, Norway

**Keywords:** abortion, Roma women, Norway, reproductive health equity, WHO framework, qualitative research

## Abstract

**Background:**

Access to abortion is a core component of sexual and reproductive health and rights (SRHR). In Norway and most European countries, abortion is legal on request. Yet, legal entitlement alone does not ensure equitable or person-centered care. Migrant women, including Roma women from Eastern Europe, often face structural and informational barriers when navigating abortion and related services.

**Objectives:**

This study explored Roma women’s experiences of abortion care, counselling, and follow-up in light of the World Health Organization’s (WHO) Conceptual Framework for Abortion Care.

**Design:**

A qualitative, exploratory design was used.

Methods: Seven semi-structured individual interviews and one focus group discussion were conducted with 12 Roma women temporarily residing in Oslo. Reflexive thematic analysis was used to analyze the data.

**Results:**

Four interrelated themes were developed:^1^ Limited access to information and contraceptive counselling,^2^ Fragmented care and missed opportunities for continuity,^3^ Bodily integrity and consent, and^4^ Autonomy, values, and the meaning of motherhood. Participants described abortion as medically safe and respectful but constrained by linguistic barriers, reliance on informal networks for information, and a lack of post-abortion counselling or follow-up.

**Conclusions:**

The study shows that Roma women’s experiences of abortion care were shaped by limited information, lack of continuity of care, and constrained autonomy. Viewed through the WHO framework, these experiences illustrate how comprehensive abortion care remains unevenly achieved for mobile and marginalized women.

## Plain language summary

We spoke with 12 women about their experiences of abortion and reproductive health services in Norway and other European countries. Many women described the abortion procedure itself as safe and respectful. However, challenges often arose before and after the procedure. Several women did not know where to seek care and relied on friends, family members, or support organisations for information. Language differences made it difficult to understand information from health professionals, especially about contraception and what to expect after the abortion. Most women said they received little or no follow-up care after the procedure. Some felt uncertain about their physical recovery, while others did not receive clear guidance on contraception. As a result, many made decisions based on incomplete or confusing information. For women who move between countries, accessing care can also become fragmented, with services spread across different health systems. This study shows that abortion care is not only about the medical procedure itself. Access to clear information, good communication, and follow-up support are also important parts of care. Improving these areas, including providing information in relevant languages and strengthening support after the procedure, could help ensure that all women receive care that is safe, respectful, and easier to understand.

## Introduction

Access to safe abortion is an essential component of sexual and reproductive health and rights (SRHR) affirming the right of every individual to make autonomous decisions about their body. Abortion remains a common procedure; an estimated 73 million abortions occur annually globally, corresponding to 39 abortions per 1,000 women aged 15–49 years.^1,2^ Six in ten unintended pregnancies and three in ten of all pregnancies end in abortion.^1,3^ Comprehensive abortion care is defined by the WHO as the full spectrum of interventions needed to ensure quality, rights-based care across the continuum of abortion and post-abortion services. This includes access to accurate information, safe abortion procedures, post-abortion contraception, and respectful follow-up care, all delivered within an enabling legal and policy environment.^4^ While legal frameworks ensuring access to abortion have expanded, evidence shows that legalization alone does not ensure equitable access. Barriers such as cost, distance, stigma, lack of information, and provider bias continue to delay or prevent access even where it is lawful.^2,5^ Women and girls living in poverty, those residing in rural areas, migrants, adolescents, and ethnic minorities are consistently identified as the groups most at risk of facing barriers to comprehensive abortion care.^3,5–7^

Across Europe, abortion laws are largely liberal, yet access and quality of care vary between and within countries.^8,9^ In Norway, abortion is legal on request up to 18 weeks of gestation with procedures provided free of charge for citizens within the public health system.^10^ Medical abortion is dominant and can be performed up to 18 weeks of gestation. Up to 10 weeks medical abortions are typically initiated in a hospital outpatient clinic with mifepristone, followed by home administration of misoprostol.^11^ Follow-up may occur in primary or specialist care and increasingly includes elements of remote or digital consultation. Migrant women, such as Roma women temporarily residing in Norway, face distinct challenges when navigating sexual- and reproductive health services resulting in lower use of modern contraception and worse maternal health outcomes.^12,13^ These disparities are reinforced by structural and social barriers such as limited awareness of available services, language difficulties, and deep-seated mistrust toward health institutions.^14–16^ Little is known about how these barriers shape their experiences of abortion and related care. This study explores how Roma women temporarily residing in Norway, with experiences from multiple European health systems, navigate abortion care, counselling, and follow-up guided by the World Health Organization’s (WHO) Conceptual Framework for Abortion Care.^4^

## WHO conceptual framework for abortion care

The World Health Organization Abortion Care Guideline^4^ includes a conceptual framework for abortion care (Illustration 1).

**Illustration 1.**
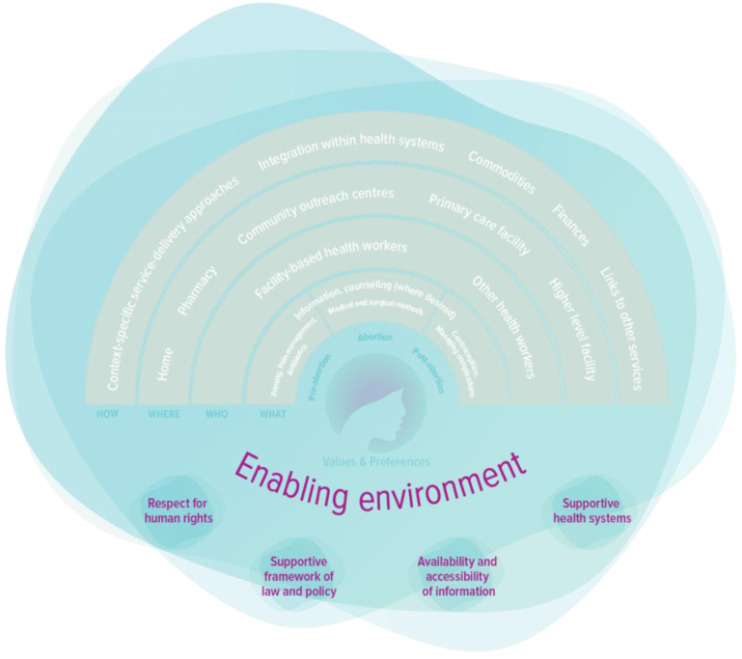
WHO conceptual framework for abortion care. Reproduced from World Health Organization. Abortion Care Guideline, second edition (4) licensed under CC BY-NC-SA 3.0 IGO.

The framework conceptualizes comprehensive abortion care as a rights-based continuum encompassing access to information, quality service provision, and an enabling legal and policy environment. This provides a rights-based structure for understanding access, quality, and equity in abortion services. The framework positions the individual’s values and preferences at the center of abortion care, recognizing that people seeking abortion must be supported to make informed, autonomous decisions in conditions of dignity, privacy, and respect. The framework is grounded in the principle that quality abortion care depends on an enabling environment that upholds human rights through a supportive legal and policy framework, ensures the availability and accessibility of accurate and culturally appropriate information, and is supported by a well-functioning health system that is universally accessible, affordable, and of good quality.^4^

## Methods

### Study design

A qualitative, exploratory study design was used to examine how Roma women residing temporarily in Norway experience abortion care, counselling, and follow-up within the public health system. WHO’s Conceptual framework for Abortion care is used to analyze and interpret the data. Semi-structured individual interviews and one focus group discussion were conducted to explore women’s perspectives on abortion, contraception, and reproductive decision-making both within the Norwegian health-care system and across Europe.

### Setting

The study was conducted in collaboration with an Oslo-based NGO providing low-threshold health and social services to Roma migrants. The partnership facilitated trust and access and ensured cultural sensitivity in recruitment and data collection. Approximately 90% of the visitors are Roma women from Romania. The initiative offers temporary accommodation, meals, and health consultations to individuals with limited access to the Norwegian welfare system.

### Participants and recruitment

Participants were adult Roma women aged 18–50 years from Romania who were temporarily residing in Oslo. Inclusion criteria were women who identified at Roma, who were migrants and aged 18-50 years old. A purposive and convenience sampling strategy was used to recruit participants through NGO networks.^17,18^ This approach ensured variation in age, parity, and prior contact with the health systems in different countries. Recruitment was facilitated by NGO staff and a bilingual Romanian nurse employed with the NGO, who served as cultural mediator and interpreter. Informational posters were displayed in Norwegian and Romanian, and staff verbally introduced the study to potential participants due to low literacy rates. No prior relationship was established between the interviewer and participants. Participants were informed about the purpose of the study and the researchers’ roles prior to participation. No participants withdrew from the study after consenting.

### Data collection

Data was collected between September and November 2024 through seven semi-structured individual interviews and one focus-group discussion (total = 12 participants). Interviews lasted 30–90 minutes and were conducted in a private consultation room at the NGO facility. All interviews were conducted by the last author with interpretation support from the Romanian nurse who also served as cultural mediatorAn interview guide (supplementary file 1) was used which covered topics such as knowledge and attitudes toward contraception, experiences with reproductive health services, and perceptions of abortion and decision-making autonomy. The interview guide was informed by key domains from the WHO Conceptual Framework for Abortion Care, such as information, quality of care, and autonomy, while remaining open to participants’ own priorities and experiences In line with reflexive thematic analysis, the study did not aim for data saturation; rather, sample size was guided by the depth and relevance of the data (information power), with data collection continuing until sufficient conceptual depth was achieved to address the research aim. Interviews were audio-recorded with consent, using the University of Oslo–approved Nettskjema app, translated and transcribed verbatim in Norwegian. The translated transcripts were discussed for accuracy with the bilingual Romanian nurse.

### Ethical consideration

The study was approved by the Ethical Committee in the Faculty of Medicine, the University of Oslo, the Norwegian Agency for Shared Services in Education and Research (Sikt) and reviewed by the Regional Committees for Medical Research Ethics (REC). Participants provided written informed consent prior to participation.

### Data analysis

Data were analyzed using reflexive thematic analysis.^19,20^ Each transcript was read and re-read to familiarize with the data, then initial codes were generated by the first author using NVivo.^21^ Both inductive and deductive approaches were used to stay close to the participants’ accounts while drawing on the WHO framework for interpretation. Here, efforts were made to capture participants’ own words and meanings of abortion care, while the WHO framework guided interpretation of how these experiences related to global standards for comprehensive abortion care and key domains such as information, quality of care, autonomy and health system responsiveness. Codes were grouped into preliminary themes that reflected patterns across participants, these themes were then refined through discussion among all authors; discrepant interpretations were resolved by revisiting the transcripts until consensus was reached. In the last steps final themes were articulated, supporting quotes selected and themes were written into a narrative. The researcher iteratively moved between the data, codes, and emerging themes, documenting analytic decisions through reflexive notes. The reporting of this study conforms to the COREQ checklist, supplementary file 2.^22^

All three co-authors are registered nurses and researchers with experience in migration and sexual and reproductive health. Our professional backgrounds and positionality as a non-Roma researchers likely shaped both the research focus and interpretation of the data. We recognize may have influenced participants willingness to share certain experiences as well as our analytical lens Reflexivity was maintained throughout the research processReflexive notes were written after each interview to enhance transparency and to document impressions, potential biases, and analytical insights.

## Results

12 women aged between their early 30s and late 40s participated in the interviews. All participants were highly mobile, having lived or sought health care in several European countries and had personal experience with abortion both in Norway or elsewhere in Europe. Four themes linked to the WHO conceptual framework were developed: Limited Access to Information and Contraceptive Counselling, Fragmented Care and Missed Opportunities for Continuity, Bodily Integrity and Consent and Autonomy, Values, and the Meaning of Motherhood.

### Limited access to information and contraceptive counselling

Many described uncertainty about where and how to seek abortion care. Information about services was most often received through informal networks or NGO staff rather than the formal health system.

“I didn’t know where to go. I asked at the shelter, and they told me I had to go to the hospital.” (Participant 3)

Participants consistently described limited access to accurate reproductive health information both before and after abortion. Several had never been informed about modern contraceptive methods prior to coming to Norway and learned about them only incidentally through peers or clinical encounters.

“I didn’t know anything about contraception before I got older. No one had talked to me about it.” (Participant 6)

The women explained how the health professionals occasionally offered brief explanations during hospital visits, but these were rarely understood or remembered due to linguistic barriers and the clinical focus on completing the abortion procedure.

Interviewer: “Did anyone talk to you about contraception after the abortion?”

“Yes, they explained a bit at the hospital.” (Participant 6) While another added, “No, nobody said anything afterwards.” (Participant 4).

Fear of side effects and loss of fertility as a result of contraception use was widespread. Many had heard from other women that hormonal contraception or intrauterine devices could cause bleeding or prevent future pregnancies.

“I took the pills for six months, but I felt very unwell and had my period twice a month. So I stopped.” (Participant 2)

“Maybe I would have been afraid that if I took the pill, I wouldn’t be able to get pregnant later. That would be bad, because having children is very important in our culture.” (Participant 8)

Information exchanged within female peer networks often replaced professional counselling, reinforcing both support and misinformation.

“I learned about contraception from my friends. We talked a lot about it, but everyone said something different. Some said that the IUD damages the womb, others that birth control pills make you sick. I didn’t know what was true.” (Participant 6)

These accounts indicate that the information domain of the WHO framework was only partially fulfilled. Women relied on informal networks for guidance, while language and cultural barriers limited their understanding of professional advice. This suggests that information was available but not accessible, limiting informed decision-making.

### Fragmented Care and Missed Opportunities for Continuity

Participants experienced the clinical procedure itself as safe and professionally managed in Norway. Most described staff as respectful and caring:

“They were kind. I felt safe.” (Participant 9)

“Everything went fine; I had no pain.” (Participant 4)

Once admitted to care, participants reported feeling physically safe and treated respectfully by health personnel, but the interaction was often brief and procedural:

“They help you, but it’s over quickly. There isn’t much time to talk.”(Participant 6)

Few received follow-up appointments, counselling, or support afterwards.

“They didn’t say what I should do afterwards.” (Participant 5)

“They asked if I wanted a coil, but I said no. I didn’t know if it was good for me.” (Participant 2)

Several expressed uncertainty about their physical recovery and lacked information about when to resume normal activity or sexual relations.

“I don’t know if everything was fine afterwards; I just went home.” (Participant 3)

Women’s descriptions of brief, procedure-focused encounters indicate that continuity, a key element of the WHO framework, was lacking. Abortion care was experienced as medically safe but disconnected from counselling and follow-up.

### Bodily integrity and consent

A few women described uncertainty and concern about what had been done to their bodies during abortion or related procedures, particularly those performed in other European countries. Before arriving in Norway, one woman recalled that a doctor had asked whether she wanted more children, and after she said no, he told her he would “do something” so she would not become pregnant again. She later feared she might have been sterilised without fully understanding or explicitly consenting to the intervention. Two other women reflected on the same experience in other European countires, one explained:

“I was asked by the doctor… I was in Italy… if I wanted more children, and I said no. And I assume, or I kind of think, that maybe the doctor turned my womb somehow. That’s why I haven’t become pregnant since.” (Participant 8)

These accounts, while based on participants’ recollections, illustrate uncertainty about what was done to their bodies and the communication about these procedures. Uncertainty about the procedures performed may reflect gaps in communication and informed consent in clinical encounters, highlighting limitations in the WHO framework’s rights and quality domains.

### Autonomy, values, and the meaning of motherhood

Decisions about abortion were deliberate and grounded in pragmatic considerations of family size, economic stability, and caregiving responsibilities. Abortion was not described as the rejection of motherhood but to act responsibly within difficult circumstances.

“We didn’t have money and lived in a small place. I couldn’t have another baby then.” (Participant 11)

Motherhood held strong symbolic and social value for all participants. Having children was seen as essential to identity, dignity, and belonging, influencing decisions about both contraception and abortion.

“It’s important to have children. But sometimes it’s not possible.” (Participant 7)

“Yes, I had abortions, but I have many children too. My children are my life.” (Participant 6)

The women described abortion as a considered decision shaped by their social and economic circumstances, rather than a rejection of motherhood. Their accounts illustrate how reproductive agency is exercised within structural constraints, reflecting the WHO framework’s emphasis on person-centred care that acknowledges individual values and lived realities.

## Discussion

Participants’ experiences of abortion care, counselling, and follow-up indicate how fragmented access to comprehensive abortion care extends beyond Norway, across European health systems. For Roma women who move between countries, sexual and reproductive health care often becomes a patchwork of encounters, seeking services wherever they are accessible, affordable, or perceived as safe. Fear of being denied treatment due to residency status or financial insecurity compounded uncertainty about where to seek care. Similar patterns have been documented across Europe, where Roma and other marginalized migrant women encounter institutional discrimination and limited access to preventive care despite formal legal protections.^23–25^ This transnational use of abortion services underscores that Europe’s largely liberal abortion laws are not equally accessible to all, but remains shaped by migration status, language, and systemic exclusion. From a reproductive justice perspective, access to abortion cannot be understood solely as a legal entitlement but as the ability to exercise reproductive decision-making within enabling social, economic, and structural conditions.^26^

Participants expressed limited access to evidence-based contraceptive information, uncertainty regarding available options, and persistent fears of infertility and side effects. These concerns were compounded by linguistic and cultural barriers and by the absence of systematic counselling before and after the procedure. The women often relied on informal networks for reproductive guidance rather than on professional health services. Consistent with previous European research, these findings illustrate that limited access to tailored information perpetuates misinformation and contributes to discontinuous contraceptive use and repeated abortions, particularly among migrant women.^27,28^ These findings underscore the importance of strengthening communication, informed consent practices, and cultural competence in healthcare, alongside systems that ensure accountability and equitable care delivery. The WHO conceptual framework situates comprehensive abortion care within health systems capable of providing a continuum of preventive and curative services.^4^ Comprehensive abortion care extends beyond the medical procedure to include post-abortion counselling, contraception, and psychosocial support within an integrated health system. In Norway, these elements are often fragmented across service levels, a fragmentation that has similarly been shown to hinder migrant women’s use of maternity health services.^29^ The women in this study described receiving care but little follow-up illustrating that continuity of care remains weak. These gaps can be understood as limitations in structural autonomy, where the ability to act on reproductive intentions is constrained not by formal rights alone, but by access to information, continuity of care, and supportive systems. Here, WHO recommends task-sharing as a practical approach to strengthen integration and close the gap between primary and specialist services.^30^ Training midwives, nurses, and community health workers to provide abortion and post-abortion contraception has been shown to be both safe and effective in improving service coverage and continuity.^31^ In the Norwegian context, this could mean empowering Maternal and Child Health Centres (called *helsestasjoner*), general practitioners, and migrant health teams to offer comprehensive abortion care. Moreover, collaboration with trusted NGOs that already engage Roma women can extend the continuum of care, ensuring that information, contraception, and referrals are accessible in appropriate languages.

Viewed through WHO conceptual framework for abortion care, participants’ experiences indicate gaps across multiple domains of the enabling environment. The framework situates quality abortion care around four interrelated elements; respect for human rights, a supportive legal and policy environment, the availability of accurate information, and a responsive health system, all of which were only partially fulfilled in this context. Respect for human rights was compromised by language barriers and limited information, undermining women’s ability to make informed and autonomous choices. A supportive framework of law and policy existed but lacked practical mechanisms to ensure continuity, counselling, and equity monitoring. Availability and accessibility of information were constrained, as participants relied on informal networks rather than professional counselling. Finally, the supportive health system dimension was weakened by the centralization of abortion services within hospitals, leaving little space for follow-up, psychosocial support, or community-based continuity of care (Illustration 2).

**Illustration 2.**
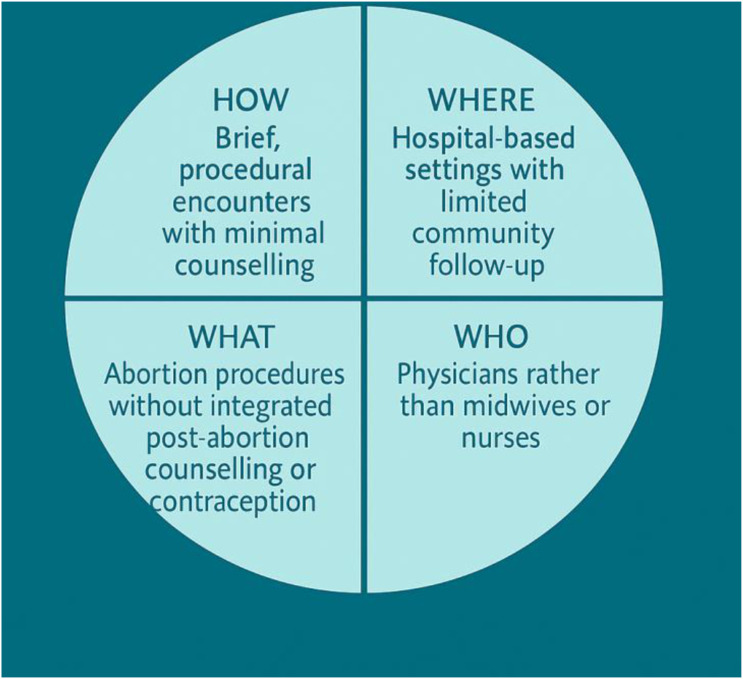
Gaps in comprehensive abortion care across WHO framework domains based on participants’ experiences.

Participants’ accounts of uncertainty regarding procedures performed during care in other European countries highlight how gaps in communication and informed consent may be experienced as violations of bodily integrity. While these accounts cannot be independently verified, they reflect uncertainty and power asymmetries in clinical encounters. These experiences should be understood in the context of documented reproductive injustices affecting Roma women in Europe, including coercive sterilisation and discriminatory practices.^32,33^ Rather than attributing intent to individual providers, they point to structural factors such as discrimination, language barriers, and unequal power relations, which have also been described among other marginalized populations.^24^

At a systems level, these findings illustrate a wider European challenge: ensuring that legal access translates into coordinated, equitable care for mobile populations.^34^ Even within countries where abortion is publicly funded and medically safe, gaps persist in the provision of comprehensive abortion care.^27^ The WHO Abortion Care Guideline emphasises that comprehensive abortion care must be integrated within health systems that provide continuous, rights-based, and person-centred services across all settings.^4^ Achieving this in Europe requires cross-border coordination and shared quality standards to ensure that services are accessible regardless of residency status. From a reproductive justice perspective, this also requires addressing the broader social and structural conditions that shape access to care, including poverty, mobility, discrimination, and legal precarity. Embedding culturally and linguistically adapted counselling within both hospital and community services alongside task-sharing among midwives and nurses would move abortion care from a reactive, procedure-focused model toward one grounded in continuity, equity, and reproductive justice.^35^

## Strengths and limitations

Using the WHO Conceptual framework for Abortion care as an analytical tool enabled systematic examination of abortion experiences beyond the clinical encounter, situating women’s narratives within legal, informational, and systemic contexts. The inclusion of Roma women, an under-researched group in the Nordic setting, contributes novel insights into how global frameworks translate into local realities. The study’s qualitative design and small sample limit generalisability. However, efforts were made to achieve variation in the sample in terms of age, parity, and experiences with different European health systems in order to ensure data richness. Data collection was conducted in collaboration with an NGO and supported by a bilingual Romanian nurse who acted as interpreter and cultural mediator. While this facilitated trust and access, it may also have influenced how participants chose to express themselves. While this enabled communication and contextual understanding, it may have influenced how participants expressed their experiences and how meaning was conveyed across languages. In addition, the presence of both the interpreter and the institutional setting may have contributed to social desirability bias, with participants potentially shaping their responses in relation to perceived expectations or relationships of trust Reflexivity was actively maintained throughout the research process, with attention to how researchers’ backgrounds and positionality may have shaped data generation and interpretation. To enhance credibility, transcripts and interpretations were discussed with the interpreter to support contextual accuracy, and triangulation of individual interviews, focus group data, and field notes was used to strengthen the data richness.

## Conclusion

This study suggests that, for Roma women temporarily residing in Norway, legal entitlement to abortion does not always translate into comprehensive or continuous care. Within the WHO abortion care framework, participants described abortion procedures as medically safe and respectful but experienced limited access to information, counselling, and post-abortion follow-up. These gaps constrained informed choice and continuity, revealing missed opportunities for comprehensive abortion care within a progressive legal context. Because many Roma women move between countries, their experiences also reflect the broader European landscape characterized by formally liberal but unevenly implemented abortion frameworks. Their reproductive care trajectories traverse multiple health systems, highlighting how structural barriers and fragmentation, rather than legal restriction alone, can limit equitable access. While these findings cannot be generalized, they point to areas where policy and practice could improve. Strengthening counselling, task-sharing among midwives and nurses, and collaboration between hospitals, municipalities, and NGOs could enhance the provision of comprehensive abortion care for mobile and marginalized populations.

## Supplemental material

Supplemental material - Between legal rights and lived realities: Roma Women’s experiences of abortion care analyzed using the WHO conceptual framework for abortion care Supplemental material for Between legal rights and lived realities: Roma Women’s experiences of abortion care analyzed using the WHO conceptual framework for abortion care by Sara Rivenes Lafontan, Frode Eick and Karoline Hasle Einang in Women's Health.

Supplemental material - Between legal rights and lived realities: Roma Women’s experiences of abortion care analyzed using the WHO conceptual framework for abortion care Supplemental material for Between legal rights and lived realities: Roma Women’s experiences of abortion care analyzed using the WHO conceptual framework for abortion care by Sara Rivenes Lafontan, Frode Eick and Karoline Hasle Einang in Women's Health.

## Data Availability

The datasets generated and/or analyzed during the current study are not publicly available due to privacy concerns. De-identified excerpts are included in the article. Requests for additional information may be considered by the corresponding author.*
