# Supplementary material for: Between legal rights and lived realities: Roma Women’s experiences of abortion care analyzed using the WHO conceptual framework for abortion care
Source: Womens Health (Lond). 2026 Jun 8;22:17455057261458329. doi: 10.1177/17455057261458329 (PMC13247287; doi:10.1177/17455057261458329)
Supplement: Supplemental material - Between legal rights and lived realities: Roma Women’s experiences of abortion care analyzed using the WHO conceptual framework for abortion care [file sj-pdf-1-whe-10.1177_17455057261458329.pdf]

Utkast intervjuguide:

1. Kan du fortelje meg litt om deg sjølv og din familie?
2. Kva tenkjer du på når du høyrer ordet «prevensjon»?
    a. I dette intervjuet brukar eg ordet «prevensjon » om å planlegge og gjere tiltak for å få born eller ikkje få born etter eige ynskje. Det vil i praksis seie at ein sjølv bestem når ein får born, og det ikkje er opp til kroppen eller andre td familien.
3. Kven pratar du med om prevensjon?
4. Korleis tenker familien din, og andre rundt deg, om planlegging av mødreskap?
5. Kjenner du til nokre typer prevensjonsmiddel, og har nokre erfaring med desse?
6. Ser du nokre ulikheiter mellom dine og andre nordmenn sine tankar kring born, familie og planlegging kring dette?
7. Opplev du noko at det er ulikheit mellom korleis du forheld deg til prevensjon når du er i Noreg og i heimlandet ditt?
8. Kva treng du frå helsevesenet i Noreg for å sikre dine behov kring prevensjon?
9. Er det noko anna du ynskjer å dele etter denne samtalen?
10. Har du nokre spørsmål til meg om denne samtalen eller temaet?

Schiță ghid interviu:

1. Poți să-mi spui puțin despre tine și familia ta?
2. La ce te gândești când auzi cuvântul „planificare familială”?
    a. În acest interviu folosesc cuvântul „planificare familială” pentru a descrie planificarea și luarea măsurilor pentru a avea sau a nu avea copii conform dorinței proprii. În practică, asta înseamnă că tu decizi când să ai copii și nu corpul sau alții, de exemplu familia.
3. Cu cine vorbești despre planificarea familială?
4. Cum gândesc familia ta și cei din jurul tău despre planificarea maternității?
5. Cunoști tipuri de metode contraceptive și ai vreo experiență cu acestea?
6. Observi vreo diferență între gândurile tale și ale altor norvegieni despre copii, familie și planificarea acestora?

7. Simți vreo diferență între modul în care te raportezi la planificarea familială când ești în Norvegia și când ești în țara ta de origine?
8. Ce ai nevoie de la sistemul de sănătate din Norvegia pentru a-ți asigura nevoile legate de planificarea familială?
9. Este ceva ce dorești să împărtășești după această conversație?
10. Ai întrebări pentru mine despre această conversație sau despre acest subiect?

Draft Interview Guide:

1. Can you tell me a little about yourself and your family?
2. What do you think of when you hear the word "family planning"?
    a. In this interview, I use the term "family planning" to refer to planning and taking measures to have or not have children according to one's own wishes. In practice, this means that you decide when to have children, and it is not up to the body or others, such as the family.
3. Who do you talk to about family planning?
4. How do your family and others around you think about planning for motherhood?
5. Do you know any types of contraceptives, and do you have any experience with them?
6. Do you see any differences between your thoughts and those of other Norwegians regarding children, family, and planning around this?
7. Do you experience any differences in how you approach family planning when you are in Norway compared to your home country?
8. What do you need from the healthcare system in Norway to ensure your family planning needs are met?
9. Is there anything else you would like to share after this conversation?
10. Do you have any questions for me about this conversation or the topic?
